# Circulating metabolites mediating the effect of psoriatic arthritis on Crohn disease risk: A mediation Mendelian randomization study

**DOI:** 10.1097/MD.0000000000047362

**Published:** 2026-01-23

**Authors:** Xia Qin, Junyang Deng, Bocheng Chen, Xi Tan, Xiaoting Xie, Guiyou Zhou, Wei Hou, Sijian Wen, Youkun Lin

**Affiliations:** a Department of Dermatology, The First Affiliated Hospital of Guangxi Medical University, Nanning, China; b The Second People’s Hospital of Nanning, The Third Affiliated Hospital of Guangxi Medical University, Nanning, China; c School of Basic Medical Sciences, Guangxi Medical University, Nanning, China; d The First Clinical Medical School, Guangxi Medical University, Nanning, China; e NHC Key Laboratory of Thalassemia Medicine, Nanning, Guangxi, China; f Guangxi Key Laboratory of Thalassemia Research, Life Sciences Institute, Guangxi Medical University, Nanning, Guangxi, China.

**Keywords:** Crohn disease, Mendelian randomization, metabolomics, psoriasis, psoriatic arthritis

## Abstract

Psoriatic arthritis (PsA) and Crohn disease (CD) are chronic immune-mediated illnesses that afflict a growing number of adults and children worldwide. Observational studies have revealed a link between PsA and CD, but the modifiable risk variables that mediate the causal effects remain unknown. We aim to look into the relationship between PsA and CD, and to see if circulating metabolites have a role in the pathophysiology of both disorders. By scanning the whole genome of a large number of people and detecting millions of genetic variation sites in the genome, we take advantage of the random assignment of genotypes in nature and use single nucleotide polymorphisms as instrumental variables to simulate the environment of randomized controlled trials, so as to infer the causal relationship between exposure factors and outcomes. Using summary statistics from genome-wide association studies of predominantly European ancestry, we used two-sample Mendelian randomization (MR) to estimate the effects of PsA on CD (1637 cases/212,242 controls; 1401 cases/461,532 controls), and two-step MR to assess the association with 1400 circulating metabolites. Genetic predisposition to PsA was associated with a higher risk of CD (pooled odds ratio, 1.00051; 95% confidence interval [CI], 1.00023–1.00078; *P* < .001). The statistical association between PsA and CD was mediated by the concentrations of homocitrulline, 5-hydroxyhexanoate, carnitine C14, gamma-glutamylthreonine, furaneol sulfate, and trans-urocanate, which accounted for 4.23% (95% CI, ‐0.68% to 9.15%, *P* = 4.58 × 10^‐2^), 7.85% (95% CI, ‐1.74% to 17.44%, *P* = 9.91 × 10^‐3^), 6.09% (95% CI, ‐1.70% to 13.88%, *P* = 3.09 × 10^‐2^), 7.45% (95% CI, ‐1.21% to 16.11%, *P* = 1.31 × 10^‐3^), 8.88% (95% CI, 0.23% to 17.54%, *P* = 8.39 × 10^‐3^), and 3.96% (95% CI, ‐0.48% to 8.40%, *P* = 2.29 × 10^‐2^) of the total effect, respectively. Our MR analysis identified significant PsA–CD associations mediated through specific metabolites, particularly the gut microbiome-derived furaneol sulfate (showing highest mediation), supporting gut–joint axis involvement. These findings establish a crucial theoretical framework for guiding clinical practice in the management of PsA and CD.

## 1. Introduction

Psoriasis (PsO) is a chronic, relapsing, immune-mediated skin disease affecting approximately 2% to 3% of the global population.^[1]^ The prevalence of PsO varies from 0.5% to 11.4% among adults and is about 1.4% in children.^[2]^ PsO is classified into several forms, including psoriasis vulgaris, guttate psoriasis, erythrodermic psoriasis and pustular psoriasis.^[3]^ Research suggested that up to 30% of people with PsO may develop psoriatic arthritis (PsA), a condition causing inflammation in the joints and surrounding tissues.^[4]^ PsA, initially characterized by Moll and Wright, is a type of seronegative inflammatory arthritis that develops in individuals with PsO.^[5]^ Clinically, patients with PsA may have an increased risk of inflammatory bowel disease (IBD), particularly Crohn disease.^[6,7]^

IBD, including Crohn disease (CD) and ulcerative colitis, is a chronic inflammatory condition of the digestive tract characterized by persistent or recurrent symptoms of varying severity.^[8]^ Globally, IBD affects approximately 3.9 million women and 3.0 million men, with prevalence steadily increasing.^[9]^

Growing evidence suggests an association between PsA and IBD, particularly CD. A population-based study reported that PsA significantly increased the risk of IBD (odds ratio [OR], 2.27; 95% confidence interval [CI], 1.86–2.77; *P* < .001).^[10]^ This risk appears even more pronounced in PsA patients compared to those with PsO alone. For example, a prospective cohort study of >170,000 women found that PsO patients had an elevated risk of CD (relative risk 3.86), while those with PsA exhibited a markedly higher risk (relative risk, 6.43; 95% CI, 2.04–20.32).^[7]^ Reported risk estimates for PsA–CD vary across studies, potentially reflecting differences in study populations or PsA phenotypic variations.

Shared mechanisms may underlie this association. Studies have identified genetic overlaps, immunological dysregulation, and gut microbiome alterations. For instance, PsA patients show reduced intestinal bacterial diversity and metabolic shifts resembling those in IBD,^[11]^ suggesting common dysbiosis-driven inflammation. However, confounding factors (such as smoking, alcohol use, stress, and immunosuppression) complicate causal interpretations.^[7,12,13]^ While some studies propose PsA as an independent risk factor for CD,^[14,15]^ further research is needed to disentangle these interactions.

Metabolomics was transforming the search for new illness biomarkers and revealing links between biomarkers and disease etiology, making it a powerful tool for precision medicine.^[16]^ It had been demonstrated that PsA can drastically alter the levels of circulating metabolites.^[17]^ Meanwhile, new research was revealing a relationship between metabolic alterations and CD.^[18]^ Metabolic factors may play a role in the development of PsA and CD. However, due to the complexities of metabolomics, there was currently no viable way to validate the metabolites associated with these diseases.

Compared to typical observational studies, which may be influenced by confounding or reverse causation, Mendelian randomization (MR) is a more trustworthy method for understanding how modifiable exposures influence specific characteristics.^[19]^ To better understand the causal link between PsA, CD, and circulating metabolites, we used a two-step MR. Our MR analysis identified significant PsA–CD associations mediated through specific metabolites. This was the first study to genetically demonstrate that PsA causes the development of CD comorbidity via circulating metabolites.

## 2. Methods

### 2.1. Study design

**Figure 1.**
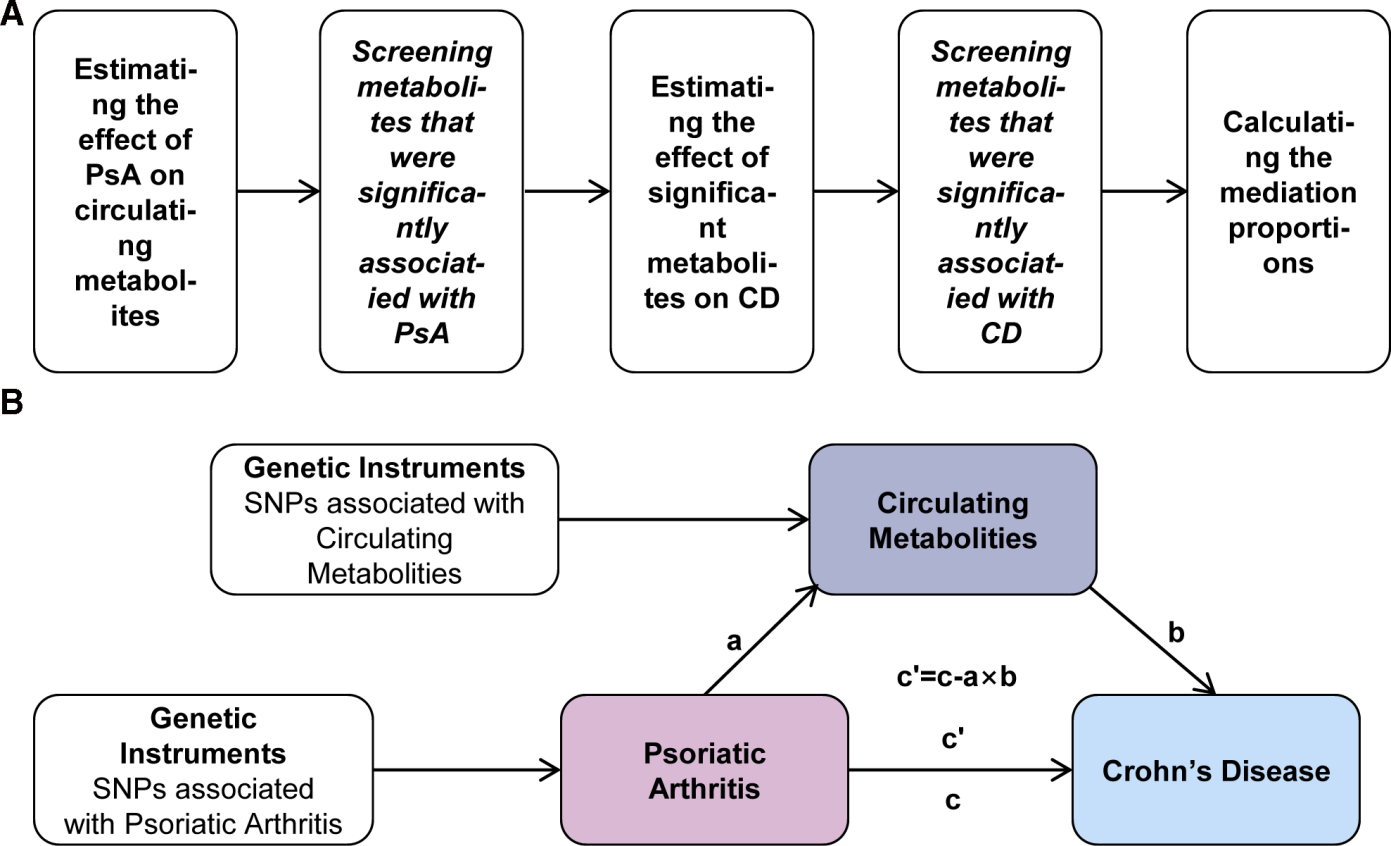
Overview of the study design. (A) The flow diagram of conducting the two-step MR step by step, which involved the selection of circulating metabolites. (B) The framework of the two-step MR. c: represents the total effect; ab: represents the mediation effect (or indirect effect); c′ represents the direct effect. CD = Crohn disease; MR: Mendelian randomization; PsA = psoriatic arthritis; SNP = single nucleotide polymorphism .

Figure 1 depicted the study design. A two-sample MR research was conducted to assess the bidirectional causality between PsA and CD. Furthermore, a mediation analysis using a two-step MR approach was carried out to see if circulating metabolites may moderate the causal pathway from PsA to CD (Fig. 1B). This study followed the Strengthening the Reporting of Observational Studies in Epidemiology Using Mendelian Randomization guidelines.^[20]^

### 2.2. Genome-wide association (GWAS) data for PsA and CD

PsA summary data were received from the Integrative Epidemiology Unit’s Open GWAS Project (https://gwas.mrcieu.ac.uk/). GWAS IDs of PsA and CD were finn-b-L12_PSORI_ARTHRO and ukb-b-8210, respectively. The GWAS meta-analysis of PsA included 1637 PsA cases and 212,242 control cases, for a total of 16,380,462 single nucleotide polymorphisms (SNPs). The GWAS meta-analysis of CD included 1401 CD cases and 461,532 control cases, for a total of 9851,867 SNPs. We extracted SNPs by analyzing the vcf files available on this platform. In this study, there was no sample overlap between the GWAS datasets for PsA and CD. The PsA GWAS data were obtained from the FinnGen study, a large-scale genomics initiative that analyzed over 500,000 Finnish biobank samples. This project represents a collaboration between Finnish research institutions and biobanks with international industry partners. In contrast, the CD GWAS data were derived from the UK Biobank, a major biomedical database and research resource containing de-identified genetic, lifestyle, and health information along with biological samples from 500,000 UK participants. The 2 datasets exhibit significant geographical differences in sample origins, precluding any possibility of sample overlap.

### 2.3. GWAS data for circulating metabolites

We applied the most recent and comprehensive GWAS datasets available for the human metabolome.^[21]^ Researchers reviewed data on 1091 blood metabolites and 309 metabolite ratios from the Canadian Longitudinal Study on Aging cohort, which included 8299 persons and about 15.4 million SNPs. The entire GWAS summary statistics for the 1400 biomarkers were made available.

### 2.4. Selection of genetic instruments

We identified SNPs of genome-wide significance (*P* < 1 × 10^‐5^). The SNPs were clumped using a 500 kb window and an linkage disequilibrium (LD) criterion of *r*^2^ < 0.01. LD values were assessed using 1000 Genomes Project data from European samples.^[22]^ Palindromic and ambiguous SNPs were excluded.^[23]^ The instruments’ strength was evaluated using *F* statistics, and weak instrumental variables (IVs) (*F*-statistics < 10) were excluded to prevent bias in the MR analysis.^[24]^

### 2.5. Statistical analysis

#### 2.5.1. Effect of PsA on CD by the MR analysis

We used two-sample MR analyses to investigate the causal links between PsA and CD. The primary causal inference approach used was inverse-variance weighted (IVW) regression with a fixed effects model.^[25]^ We then used MR-Egger, weighted median, weighted mode, and simple mode methods to supplement and improve the trustworthiness of the data. When only one genetic instrument was available, we employed the Wald ratio for MR analysis. To ensure the robustness of our findings, we did a sensitivity analysis to account for potential confounding factors influencing the exposure–outcome relationships, as shown below. We conducted a PhenoScanner search,^[26,27]^ to evaluate all known phenotypes connected to the genetic instruments used in our investigations.

#### 2.5.2. Mediation MR analysis linking PsA with CD via circulating metabolites

We also performed a mediation analysis using a two-step MR design to see if the levels of specific metabolites may mediate the causal pathway from PsA to CD. We initially used two-sample MR to determine PsA’s effect on circulating metabolites (a in Fig. 1B). Second, we used two-sample MR to assess the influence of metabolites having statistically significant relationships with PsA on CD (b in Fig. 1B). The total effect gained in previous MR analysis (c in Fig. 1B) can be decomposed into an indirect effect (through mediators, a × b in Fig. 1B) and a direct effect (without mediators, c′ in Fig. 1B) effect.^[28]^

By dividing the indirect effect by the total effect, we could determine the percentage mediated by the mediating effect. Concurrently, the delta approach was utilized to compute 95% CIs.

We searched for mediators of the relationship between PsA and CD using the following criteria: PsA and the mediator had a causal relationship, and the effect of PsA on the mediator should be unidirectional, because bidirectionality between them may affect the validity of the mediation analysis. The mediator and CD have a consistent causal connection, with or without PsA correction. According to current scientific research, the associations between PsA and the mediator, as well as the associations between the mediator and CD, should be in the same direction.

### 2.6. Sensitivity analysis

With the aid of funnel plots and Cochran *Q* statistic, the heterogeneity between SNPs was evaluated,^[29]^ and a *P* value of <.05 would be considered significant heterogeneity.^[30]^ The MR-Egger intercept^[31]^ methods were used to identify horizontal pleiotropy. The weighted median method provides a robust estimate of the effect, when more than half of IVs are valid.^[32]^ We removed any identified outliers and reevaluated the MR causal estimates. To ensure the stability of the results in the presence of heterogeneity after outlier removal, we utilized a random effects model, which was more robust to weaker associations between SNPs and exposure. Additionally, we conducted a “leave-one-out” sensitivity analysis, where the MR is left out individually to identify potentially significant SNP. R version 4.3.2 (R Foundation for Statistical Computing, Vienna, Austria) with the two-sample MR package was used for all statistical analyses.^[23]^ Statistical significance was defined as *P* value <.05.

## 3. Results

### 3.1. Effect of PsA on CD

**Figure 2.**
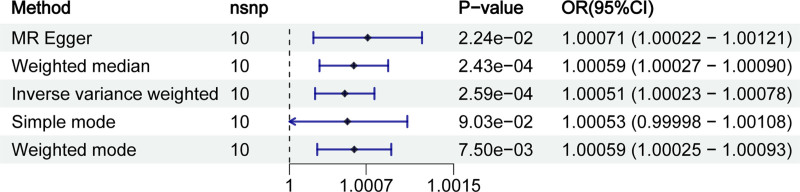
MR estimates (based on IVW) of the effect of PsA on CD. CD = Crohn disease; IVW = inverse-variance weighted; MR = Mendelian randomization; PsA = psoriatic arthritis.

After removing confounding factors, a total of 15 SNPs were chosen as PsA IVs (Table S1, Supplemental Digital Content, https://links.lww.com/MD/R209). The main results were obtained using the radial IVW approach with updated second-order weights after the final iteration and are shown as ORs with 95% confidence intervals. Our MR analysis revealed a statistically significant association between PsA and increased CD risk (IVW: OR = 1.00051 [95% CI, 1.00023–1.00078], *P* < .001) (Fig. 2 and Table S2, Supplemental Digital Content, https://links.lww.com/MD/R209), while reverse MR analysis showed no evidence for CD influencing PsA risk (*P* = .25) (Table S3, Supplemental Digital Content, https://links.lww.com/MD/R209). There was no heterogeneity or horizontal pleiotropy amongst instruments when examining the influence of PsA on CD.

### 3.2. Effect of PsA on circulating metabolites and CD via the mediation MR analysis

**Figure 3.**
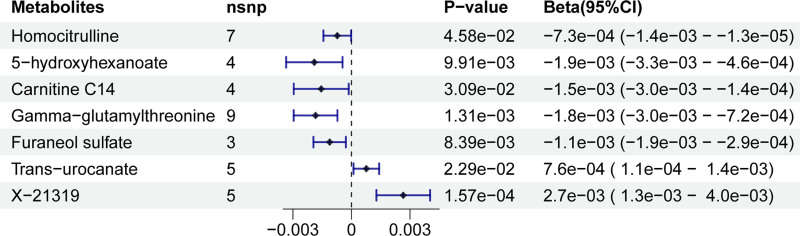
Forest plot of the causal effects of PsA-related metabolites on the risk of CD derived from the IVW method. CD = Crohn disease; IVW = inverse-variance weighted; PsA = psoriatic arthritis.

**Figure 4.**
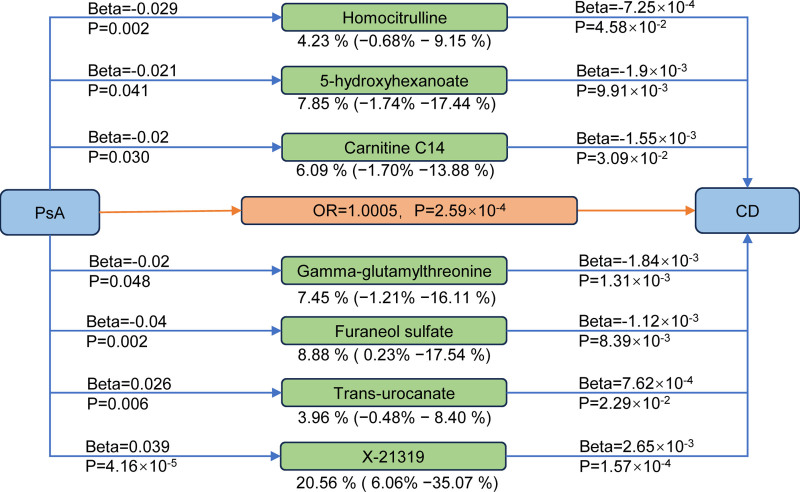
The mediation effects of metabolites on the association between PsA and CD. CD = Crohn disease; PsA = psoriatic arthritis.

A two-step MR was performed to see if the levels of specific metabolites may mediate the causal pathway from PsA to CD. We first calculated the effect of PsA on 1400 circulating metabolites and showed that 176 metabolites were significantly linked with PsA (IVW: *P* < .05, Cochran *Q* statistic: *P* > .05 and pleiotropy: *P* > .05) (Tables S4 and S5, Supplemental Digital Content, https://links.lww.com/MD/R209). Secondly, we investigated the influence of 176 circulating metabolites that were substantially linked with PsA on CD and the results were showed in Table S6, Supplemental Digital Content, https://links.lww.com/MD/R209 (IVW: *P* < .05, Cochran *Q* statistic: *P >* .05 and pleiotropy: *P* > .05). After removing the metabolites in the opposite direction of the first and second steps, 7 metabolites were obtained (Fig. 3). We observed an indirect effect of PsA on CD through homocitrulline, 5-hydroxyhexanoate, carnitine C14, gamma-glutamylthreonine, furaneol sulfate, and trans-urocanate, with a mediated proportion of 4.23% (95% CI, ‐0.68% to 9.15%, *P* = 4.58 × 10^‐2^), 7.85% (95% CI, ‐1.74% to 17.44%, *P* = 9.91 × 10^‐3^), 6.09% (95% CI, ‐1.70% to 13.88%, *P* = 3.09 × 10^‐2^), 7.45% (95% CI, ‐1.21% to 16.11%, *P* = 1.31 × 10^‐3^), 8.88% (95% CI, 0.23% to 17.54%, *P* = 8.39 × 10^‐3^), 3.96% (95% CI, ‐0.48% to 8.40%, *P* = 2.29 × 10^‐2^) of the total effect, respectively (Fig. 4). We observed an indirect effect of PsA on CD through an unknown metabolite-X 21319, with a mediated proportion of 20.56% (95% CI, 6.06% to 35.07%, *P* < .001) (Fig. 4). There were no evidence of heterogeneity and no horizontal pleiotropy among these associations (File S1, Supplemental Digital Content, https://links.lww.com/MD/R211).

## 4. Discussion

Numerous studies had found a high incidence of co-morbidities between PsA and CD.^[33–35]^ Research had shown that IL-23 and IL-17A interact in a pathogenic manner in PsA, and differential methylation sites in peripheral blood can distinguish between PsA and PsO, signaling that DNA methylation could be a promising predictive biomarker for PsA.^[36]^ The IL-17/IL-23 axis was critical for PsA development and maintenance^[37]^ In patients with spondyloarthritis, such as PsA, group 3 innate lymphocytes cells raise levels of IL-17A and IL-22, leading to chronic inflammation and gut barrier damage.^[38]^ Additionally, CD8+ T cells played a major role in the pathogenesis of PsA, as expected given the link of PsA with the HLA-I allele.^[37]^ In CD, it had been proposed that dendritic cells may lose their ability to induce tolerant regulatory T cells.^[39]^ CD was characterized by an imbalance between effector T cells (primarily Th1 or Th17 cells) and natural regulatory T cells (secreting IL10, interleukin 35) derived from the thymus (nTreg) and inducer cells (iTreg) such as Tr1, Th3, and iTr35.^[40]^ Furthermore, GWAS provided support for the imbalance model by associating key sites of differentiation and CD with Tregs (e.g., IL10, IL2RA, SMAD3) and Th1 and Th17 (e.g., CPEB4).^[41]^ The bridge between PsA and IBD may be the Th17-mediated gut microbiome.^[42]^ The causal relationship between the PsA and CD is still debated. Our MR analysis revealed a statistically significant association between PsA and increased CD risk (IVW: OR = 1.00051; 95% CI, 1.00023–1.00078, *P* < .001), which was consistent with previous findings.^[14,15]^ However, the mediating factors causing this effect still need to be further explored.

PsA and its associated metabolites are frequently reported.^[43]^ A growing body of research suggested that people with PsA have metabolic abnormalities compared to the general population.^[44]^ Our MR analysis not only genetically demonstrates the association between PsA and the risk of CD (IVW: OR = 1.00051; 95% CI, 1.00023–1.00078, *P* < .001), but also precisely identifies potential modifiable metabolic intermediates that may mediate this association.

Homocitrulline belonged to a class of citrulline, which was a nonprotein amino acid that, in the human body, was derived from the amount produced primarily in the cells of the small intestine.^[45]^ By using a synthetic citrulline-rich peptide, joint epitope determinant (referred to here as citrullinated joint epitope determinant), and its homocitrullinated counterpart, Patrick Lac et al found that antibodies targeting citrullinated joint epitope determinant and homocitrullinated counterpart were commonly detected in the sera of rheumatoid arthritis (RA) patients but were rarely present in healthy individuals or patients with systemic lupus erythematosus or PsA.^[46]^ This result was consistent with ours, indicating that PsA can reduce the levels of homocitrulline. Both posttranslational modification (PTM)-directed memory B cells, which exhibited extensive cross-reactivity to all 3 PTM antigens (homocitrulline, isocitrulline, and ethocoolysine), and PTM-directed plasmablasts showed high expression of CXCR3, a chemokine receptor abundant in arthritic joints.^[47]^ Other studies had shown that the levels of citrulline in Crohn patients was lower than that in the control group,^[48]^ which was consistent with our MR study. We surmised that the levels of homocitrulline can be used to distinguish RA from PsA, and low levels of homocitrulline may increase the risk of CD.

Gamma-glutamylthreonine had an amino acid structure similar to glutamine and was synthesized by the amino acid metabolic pathway from the precursor’s glutamate, cysteine, and threonine. In our MR study, we found that PsA is able to reduce the levels of gamma-glutamylthreonine, and the low levels of gamma-glutamylthreonine can contribute to the risk of CD. Depletion of glutathione levels can significantly hinder an organism’s capacity to combat oxidative stress, resulting in cell damage and potential organism demise.^[49]^ The level of gamma-glutamylthreonine is reduced, leading to the reduction of intestinal antioxidant defense capacity. Reactive oxygen species are more likely to cause tissue damage in IBD, thereby increasing the risk of CD.^[50]^ Therefore, PsA may increase the risk of CD by reducing the levels of gamma-glutamylthreonine, which leads to a decrease in the ability of cells to resist oxidative stress.

Lipids were known to play an indisputable role in psoriatic arthritis.^[51]^ Genetic studies had confirmed that in some autoimmune diseases, lipid mediators were not only the result of oxidative stress and inflammation but also played an important role in regulating these processes.^[52]^ In patients with PsO or PsA, several research groups had found many lipid changes, including changes in the concentration of total cholesterol, low-density lipoprotein cholesterol, triglycerides, or lipoprotein Lp (a), or decreases in the concentration of high-density lipoprotein cholesterol.^[53–55]^ Patients with PsA may exhibit abnormalities in apolipoprotein or oxidized low-density lipoprotein levels, with the severity of PsA potentially linked to higher concentrations of small, dense LDL.^[56]^ A study also found that people with CD had significantly lower lipid levels in their bodies than people with CD who were in remission.^[57]^ In our MR study, we found that PsA can reduce levels of lipid metabolites (carnitine C14 and 5-hydroxyhexanoate). We found that PsA can increase the risk of CD by reducing the levels of 5-hydroxyhexanoate and carnitine C14. This is the first report on the causal relationship between PsA, 5-hydroxyhexanoate, carnitine C14, and CD.

In our MR analysis, we found that PsA reduces the levels of furaneol sulfate, and low levels of furaneol sulfate were associated with an increased risk of CD. Furaneol sulfate showed a robust mediation effect (8.88%, 95% CI 0.23–17.54), suggesting it actively contributes to the PsA–CD link. Furaneol sulfate is a phase II metabolite formed via sulfation of furaneol, a process that can modulate its biological activity, but studies on furaneol sulfate are still limited to date. A study found that gut microbiota-derived sulfur metabolic pathways (e.g., sulfate-reducing bacteria) in IBD patients are associated with disease activity, indirectly supporting the potential role of sulfur-containing metabolites (such as furaneol sulfate).^[58]^ A mediated analysis also suggested that furaneol sulfate may bridge the causal relationship between intestinal flora and juvenile idiopathic arthritis.^[59]^ According to our results, furaneol sulfate may also serve as a bridge between PsA and CD, and this bridging effect may be mediated through the gut microbiot.^[60]^ Its high mediation proportion aligns with the shared gut–joint axis in PsA–CD comorbidity.

Our MR study also showed that PsA could increase levels of trans-urocanate. Histidine can be decomposed by histidinase (histidine lyase) to produce ammonia and trans-urocanate.^[61]^ Research has found that patients with PsA exhibit lower serum histidine levels,^[62]^ which may lead to reduced production of its metabolic product trans-urocanate. This finding indirectly supports that trans-urocanate only plays a limited mediating role in PsA-associated CD. In another study, low histidine concentrations were found in RA patients, which may be explained by excessive decarboxylation of histidine in the joints.^[63]^ This could potentially explain the decreased levels of serum histidine seen in other inflammatory synovial conditions, such as PsA.^[62]^ A study discovered that trans-urocanate was detected in the serum and colonic cavity of conventional mice, but not in germ-free mice.^[64,65]^ Our MR study demonstrated that PsA elevates CD risk through upregulation of trans-urocanic acid levels in genetic variants, albeit with a limited mediating effect.

Our MR analysis identified significant heterogeneity in the mediation effects of specific metabolites linking PsA to CD. The gut-derived metabolite furaneol sulfate demonstrated the strongest mediation effect (8.88%), followed by gamma-glutamylthreonine (7.45%), 5-hydroxyhexanoate (7.85%), and carnitine C14 (6.09%). In contrast, homocitrulline and trans-urocanate showed more modest mediation proportions (4.23% and 3.96%, respectively).

These findings provide 3 key translational insights: *risk stratification*: the identified metabolites, particularly furaneol sulfate, may serve as predictive biomarkers for CD risk in PsA patients; *mechanistic understanding*: the observed mediation hierarchy (furaneol sulfate > carnitine C14 > trans-urocanate) suggests gut-derived systemic mediators play a more prominent role than tissue-specific factors; *therapeutic potential*: these metabolic intermediates represent promising targets for preventive interventions, including dietary modifications and pharmacological modulation.

The observed heterogeneity in mediation effects likely reflects both genuine biological differences among metabolites and inherent methodological limitations of our analysis. While our findings highlight the importance of gut–systemic pathways, we cannot exclude potential tissue-specific concentration effects, underscoring the need for future localized metabolomic profiling studies.

## 5. Strengths and limitations

This represents the first MR study investigating the causal relationships between PsA, circulating metabolites, and CD manifestations. While MR provides robust causal inference by minimizing confounding, several important limitations warrant consideration: first, inherent MR constraints: the study could not fully disentangle direct versus indirect effects of metabolites due to MR’s inherent limitation in establishing intermediate pathways. While our analysis identifies metabolite-disease associations, the biological mechanisms (e.g., whether effects are mediated through inflammation, immune regulation, or other pathways) remain unclear. MR estimates reflect lifetime exposure effects rather than acute or time-specific impacts, potentially limiting clinical translatability. Second, methodological considerations: Potential weak instrument bias and horizontal pleiotropy may persist despite sensitivity analyses (MR-Egger, weighted median). Incomplete metabolite coverage due to unannotated or missing GWAS data limits pathway interpretation. Population stratification and LD bias could influence results despite genetic clumping. The linearity assumption may not capture complex, nonlinear biological relationships. Third, biological interpretation: while we identified significant metabolite-disease associations, tissue-specific effects (e.g., gut vs systemic metabolite actions) could not be assessed due to GWAS data limitations. Potential bidirectional effects between metabolites and disease states remain unexplored in this unidirectional MR framework. These limitations highlight the need for complementary approaches (e.g., mediation analysis, experimental studies) to validate and mechanistically explain our findings. Nevertheless, this study provides foundational evidence for metabolite-driven disease mechanisms in PsA and CD. While PsA and axial spondyloarthritis share clinical features as spondyloarthropathies, this study specifically examines the PsA–CD relationship through the lens of metabolic interactions, without direct comparison to axial spondyloarthritis due to fundamental differences in disease definitions and study objectives.

## 6. Conclusions

Our MR study revealed a statistically significant genetic link between PsA and CD risk, potentially mediated by circulating metabolites such as homocitrulline, 5-hydroxy hexanoate, carnitine C14, gamma-glutamyl threonine, and furaneol sulfate. While these findings suggest a possible metabolic influence on disease interplay, they remain hypothetical and require experimental and clinical validation. No current evidence supports metabolite supplementation for PsA or CD prevention, and our results should not be interpreted as a clinical recommendation. Instead, these insights highlight novel pathways for future mechanistic research and potential therapeutic exploration in PsA-associated CD.

## Acknowledgments

The authors would like to express the gratitude to other authors, all the people who provide help and support in the writing process. This manuscript is not under review with any other journal now and has not been published in any other journal previously.

## Author contributions

**Conceptualization:** Xia Qin.

**Data curation:** Xia Qin.

**Formal analysis:** Xia Qin.

**Funding acquisition:** Youkun Lin.

**Investigation:** Xia Qin, Junyang Deng.

**Methodology:** Xia Qin.

**Project administration:** Xia Qin.

**Resources:** Xia Qin.

**Software:** Bocheng Chen, Junyang Deng.

**Supervision:** Youkun Lin, Sijian Wen.

**Validation:** Xia Qin.

**Visualization:** Xia Qin.

**Writing – original draft:** Xia Qin, Bocheng Chen, Junyang Deng, Xiaoting Xie, Guiyou Zhou, Xi Tan, Wei Hou.

**Writing – review & editing:** Xia Qin.

## Supplementary Material




