# Supplementary material for: Circulating metabolites mediating the effect of psoriatic arthritis on Crohn disease risk: A mediation Mendelian randomization study
Source: Medicine (Baltimore). 2026 Jan 23;105(4):e47362. doi: 10.1097/MD.0000000000047362 (PMC12851653; doi:10.1097/MD.0000000000047362)
Supplement: Supplementary file 2 [file medi-105-e47362-s002.docx]

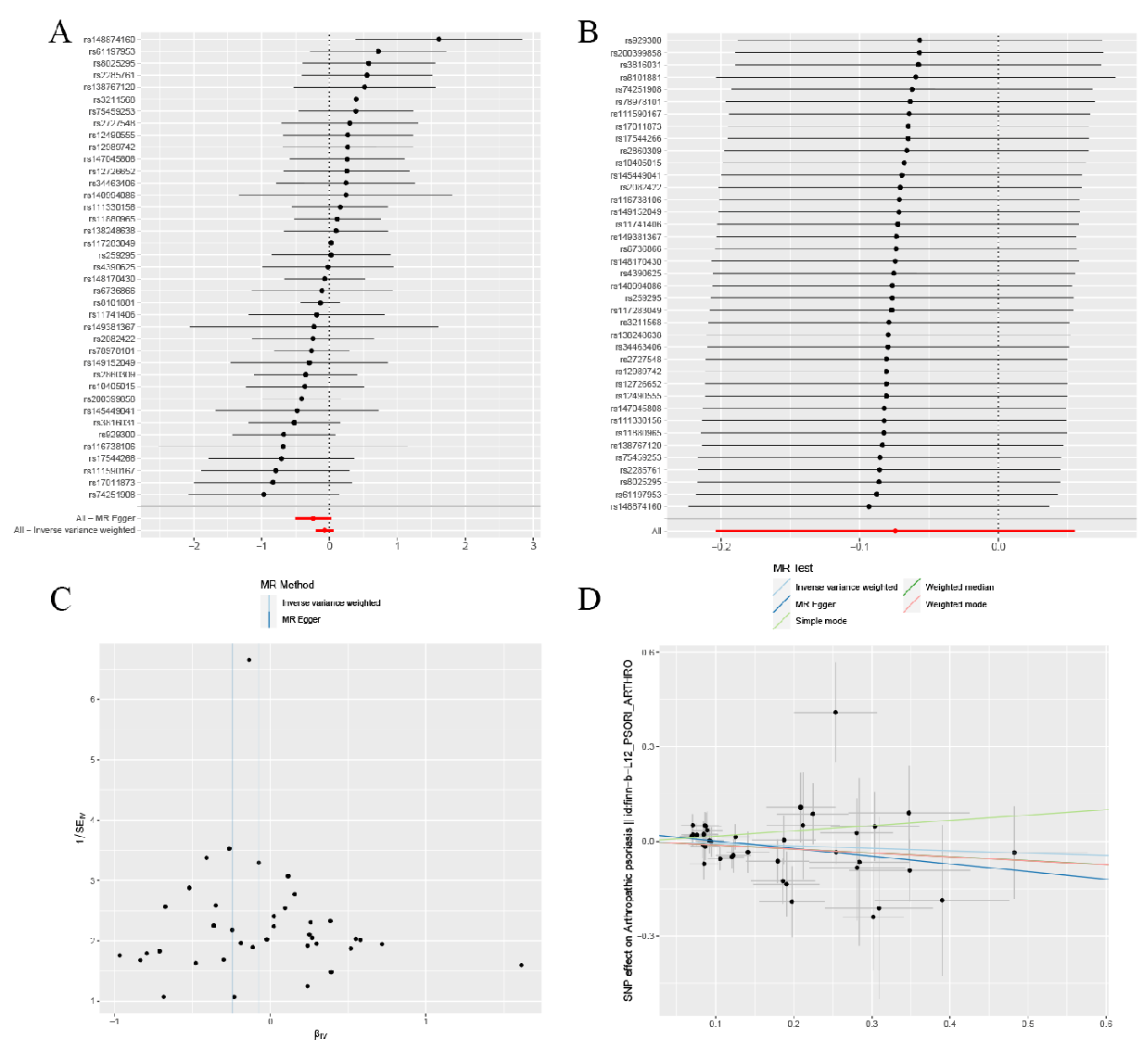
**Figure S1.** Mendelian Randomization (MR) Analysis of homocitrulline with Heterogeneity and Horizontal Pleiotropy Assessments. (A) Forest plot of individual SNP effect estimates for Homocitrulline; (B) Leave-one-out sensitivity analysis evaluating the stability of MR estimates; (C) Funel plot assessing potential heterogeneity across genetic instruments; (D) Scatter plot illustrating the association between SNP-exposure and SNP-outcome effects.


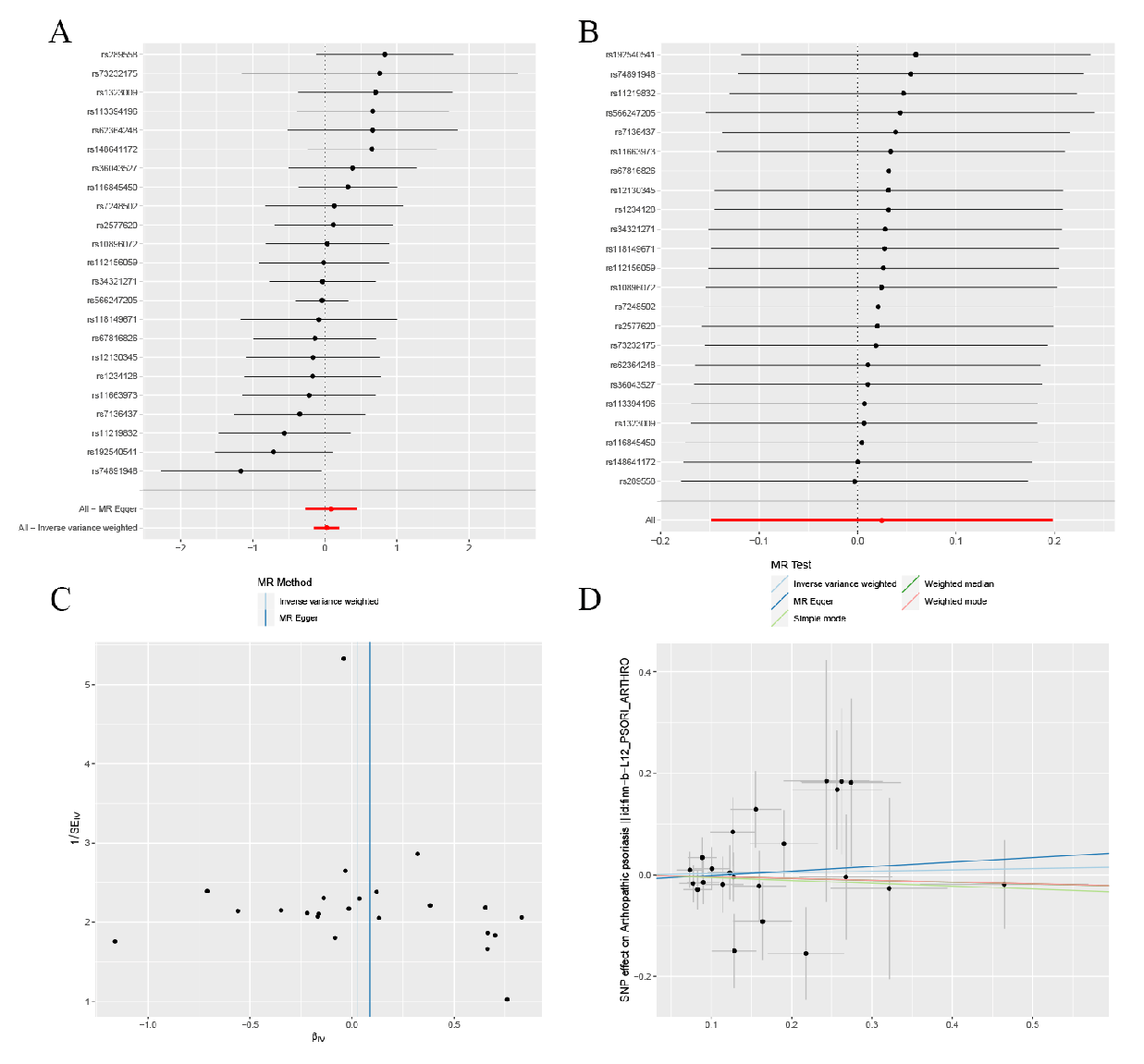
**Figure S2.** Mendelian Randomization (MR) Analysis of 5-hydroxyhexanoate with Heterogeneity and Horizontal Pleiotropy Assessments. (A) Forest plot of individual SNP effect estimates for 5-hydroxyhexanoate; (B) Leave-one-out sensitivity analysis evaluating the stability of MR estimates; (C) Funel plot assessing potential heterogeneity across genetic instruments; (D) Scatter plot illustrating the association between SNP-exposure and SNP-outcome effects.


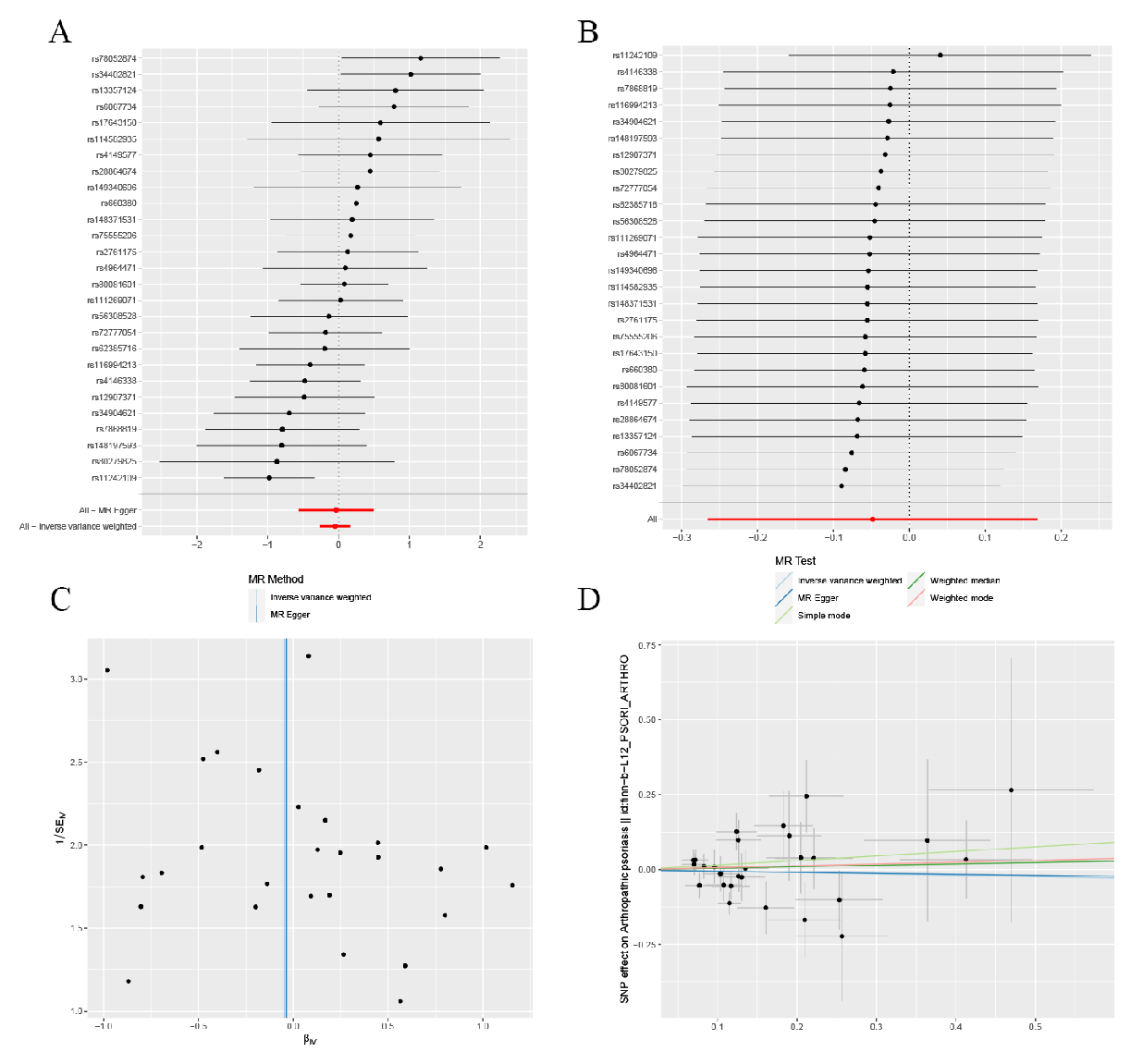
**Figure S3.** Mendelian Randomization (MR) Analysis of Carnitine C14 with Heterogeneity and Horizontal Pleiotropy Assessments. (A) Forest plot of individual SNP effect estimates for Carnitine C14; (B) Leave-one-out sensitivity analysis evaluating the stability of MR estimates; (C) Funel plot assessing potential heterogeneity across genetic instruments; (D) Scatter plot illustrating the association between SNP-exposure and SNP-outcome effects.


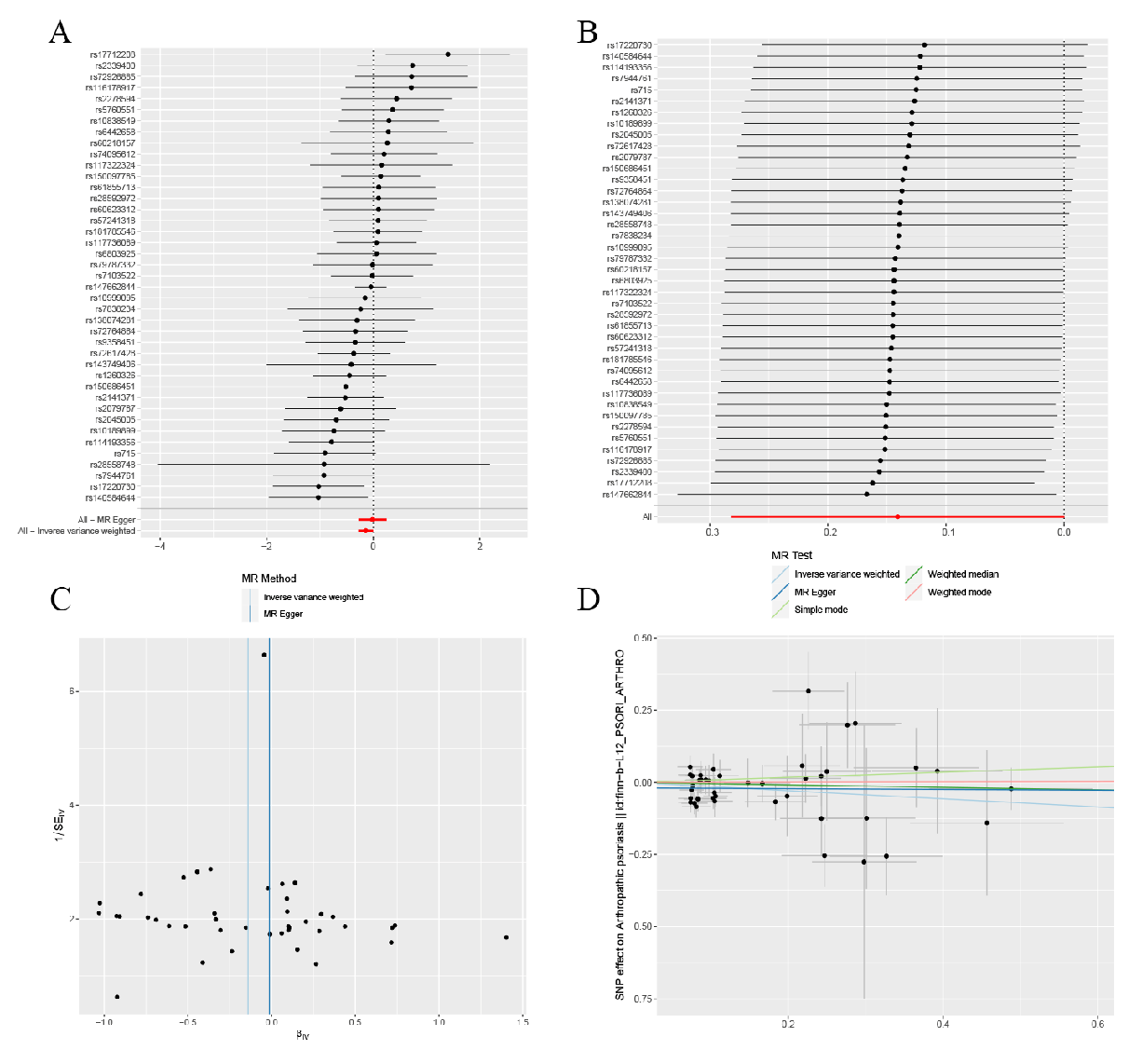
**Figure S4.** Mendelian Randomization (MR) Analysis of gamma-glutamylthreonine with Heterogeneity and Horizontal Pleiotropy Assessments. (A) Forest plot of individual SNP effect estimates for Gamma-glutamylthreonine; (B) Leave-one-out sensitivity analysis evaluating the stability of MR estimates; (C) Funel plot assessing potential heterogeneity across genetic instruments; (D) Scatter plot illustrating the association between SNP-exposure and SNP-outcome effects.


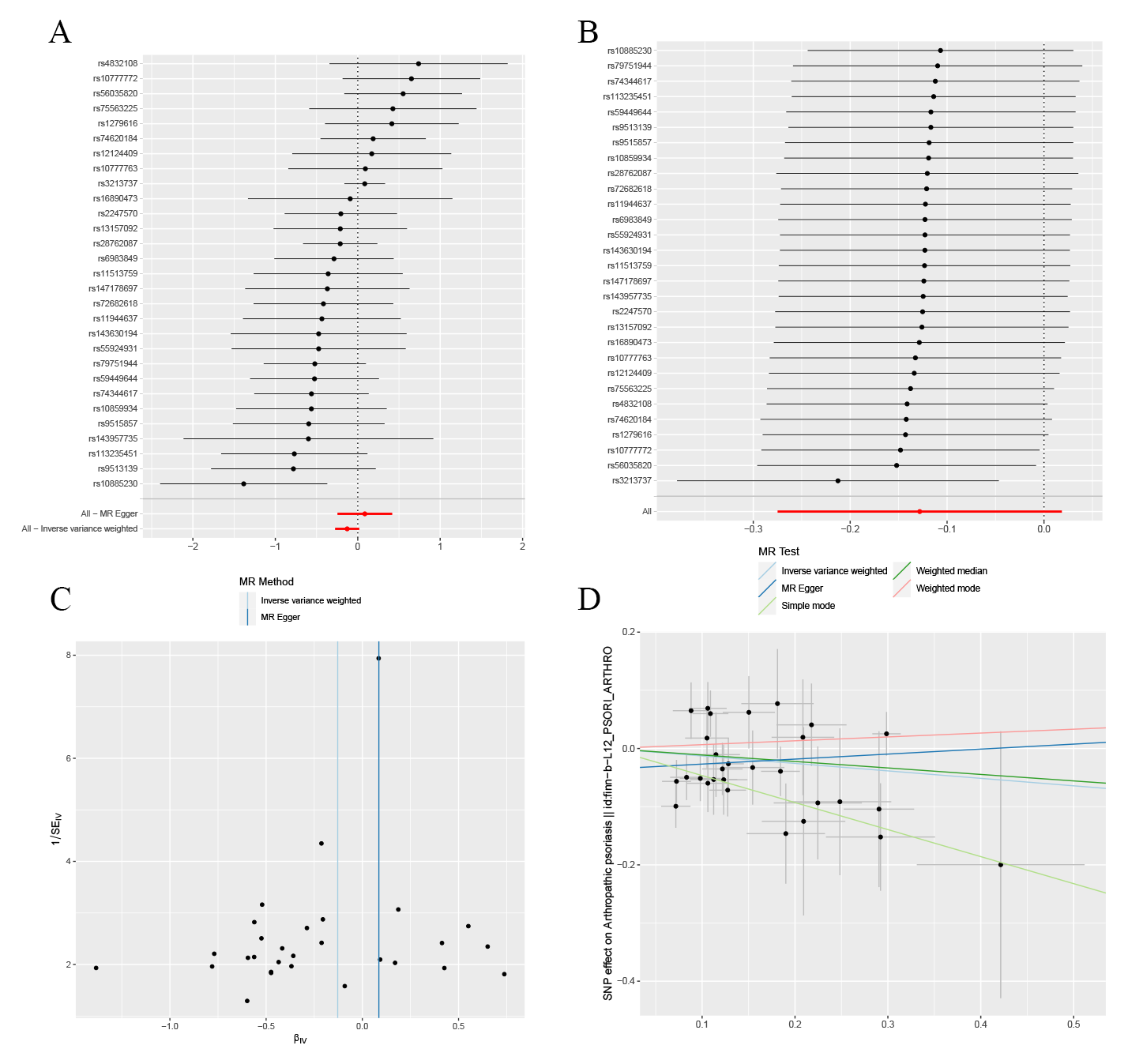
**Figure S5.** Mendelian Randomization (MR) Analysis of furaneol sulfate with Heterogeneity and Horizontal Pleiotropy Assessments. (A) Forest plot of individual SNP effect estimates for Furaneol sulfate; (B) Leave-one-out sensitivity analysis evaluating the stability of MR estimates; (C) Funel plot assessing potential heterogeneity across genetic instruments; (D) Scatter plot illustrating the association between SNP-exposure and SNP-outcome effects.


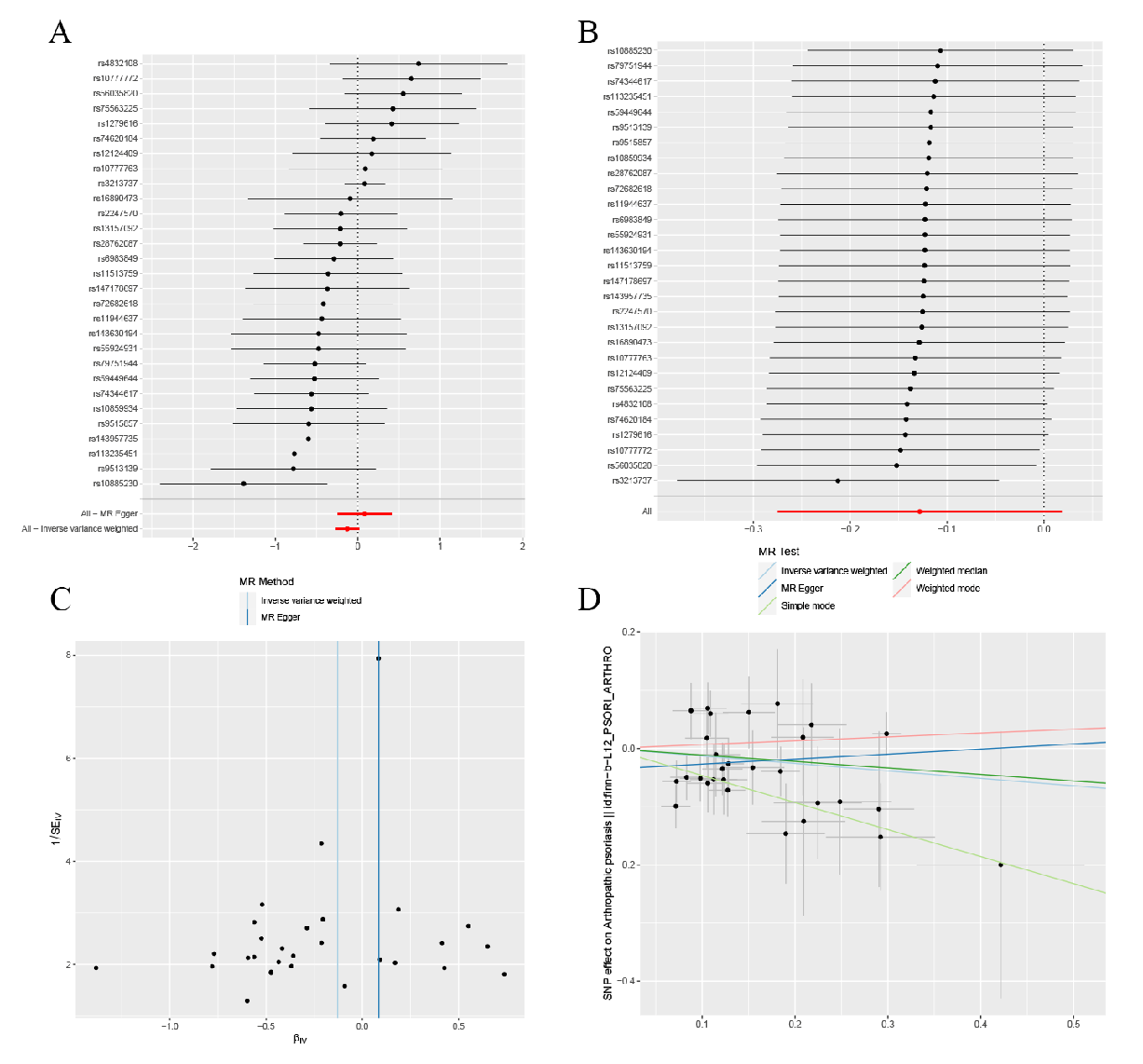
**Figure S6.** Mendelian Randomization (MR) Analysis of trans-urocanate with Heterogeneity and Horizontal Pleiotropy Assessments. (A) Forest plot of individual SNP effect estimates for Trans-urocanate; (B) Leave-one-out sensitivity analysis evaluating the stability of MR estimates; (C) Funel plot assessing potential heterogeneity across genetic instruments; (D) Scatter plot illustrating the association between SNP-exposure and SNP-outcome effects.


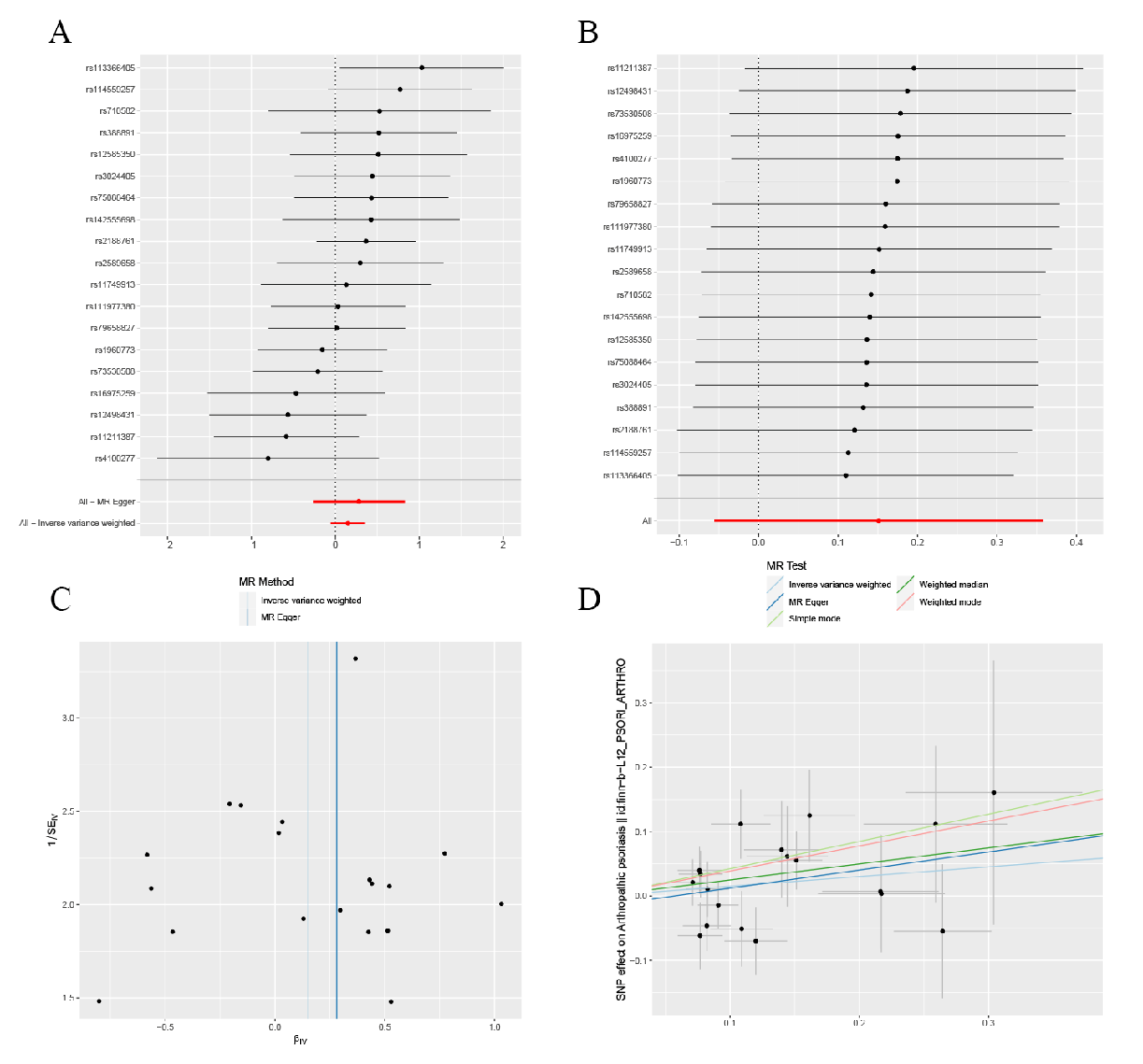
**Figure S7.** Mendelian Randomization (MR) Analysis of X-21319 with Heterogeneity and Horizontal Pleiotropy Assessments. (A) Forest plot of individual SNP effect estimates for X-21319; (B) Leave-one-out sensitivity analysis evaluating the stability of MR estimates; (C) Funel plot assessing potential heterogeneity across genetic instruments; (D) Scatter plot illustrating the association between SNP-exposure and SNP-outcome effects.
